# Associations among Ratio of Free Triiodothyronine to Free Thyroxine, Chronic Kidney Disease, and Subclinical Hypothyroidism

**DOI:** 10.3390/jcm11051269

**Published:** 2022-02-25

**Authors:** Yuji Shimizu, Shin-Ya Kawashiri, Yuko Noguchi, Seiko Nakamichi, Yasuhiro Nagata, Naomi Hayashida, Takahiro Maeda

**Affiliations:** 1 Department of General Medicine, Nagasaki University Graduate School of Biomedical Sciences, Nagasaki 852-8501, Japan; tmaeda@nagasaki-u.ac.jp; 2 Department of Cardiovascular Disease Prevention, Osaka Center for Cancer and Cardiovascular Diseases Prevention, Osaka 537-8511, Japan; 3 Department of Community Medicine, Nagasaki University Graduate School of Biomedical Sciences, Nagasaki 852-8523, Japan; shin-ya@nagasaki-u.ac.jp (S.-Y.K.); y-noguti@nagasaki-u.ac.jp (Y.N.); ynagata1961@nagasaki-u.ac.jp (Y.N.); 4 Leading Medical Research Core Unit, Nagasaki University Graduate School of Biomedical Sciences, Nagasaki 852-8523, Japan; naomin@nagasaki-u.ac.jp; 5 Nagasaki University Health Center, Nagasaki 852-8523, Japan; seiko-n@nagasaki-u.ac.jp; 6 Division of Promotion of Collaborative Research on Radiation and Environmental Health Effects, Atomic Bomb Disease Institute, Nagasaki University, Nagasaki 852-8523, Japan

**Keywords:** triiodothyronine, thyroxine, subclinical hypothyroidism, chronic kidney disease, FT3/FT4, euthyroid, demand, prevent

## Abstract

The ratio of free triiodothyronine (FT3) to free thyroxine (FT4) (FT3/FT4), a maker of peripheral thyroxin deiodination, could indicate activity of thyroid hormone. Since positive association between subclinical hypothyroidism (SCH) and chronic kidney disease (CKD) was reported, clarifying the association among FT3/FT4, SCH, and CKD could be an efficient tool to make a strategy for preventing CKD. A cross-sectional study with 1724 Japanese with normal thyroid hormone was conducted. Significant positive association between SCH and CKD was observed; the adjusted odds ratio (OR) and 95% confidence interval (95% CI) was 2.23 (1.38, 3.59). Even though, FT3/FT4 was found to be inversely associated with CKD whereas positively associated with SCH; the adjusted ORs and 95% CIs for 1 standard deviation (SD) increment of FT3/FT4 were 0.51 (0.35, 0.74) for CKD and 2.40 (1.34, 4.29) for SCH, respectively. FT3/FT4 was also found to be positively associated with SCH without CKD but not those with CKD; 1 SD increment of FT3/FT4 were 3.44 (1.72, 6.91) for SCH without CKD and 1.11 (0.40, 3.06) for SCH with CKD, respectively. Although further investigation is necessary, present study indicates that higher activity of peripheral thyroxin deiodination might have beneficial association on absence of CKD even among SCH which is positively associated with CKD.

## 1. Introduction

Two types of hormones are regarded as thyroid hormones: triiodothyronine (T3) and thyroxine (T4). Although both T3 and T4 are known as thyroid hormones, daily production of T3 by the thyroid gland is limited while T4 is produced almost daily by the thyroid gland. Most daily production of T3 originates from peripheral deiodination of T4 [1]. Only 20% of the total integrated daily production of T3 is secreted directly from the thyroid gland. The remaining 80% of T3 originates from peripheral deiodination of T4 by deiodinase [1]. The enzyme that contributes to deiodination has been found in the liver and kidney [2,3]. In contrast, T4 is produced exclusively in the thyroid gland. Therefore, active deiodination that increases conversion of free T4 (FT4) to free T3 (FT3) also increases the FT3/FT4 ratio [4]. Therefore, peripheral deiodination activity could be evaluated by the ration of FT3 to FT4 (FT3/FT4), which reflects the conversion of FT4 to FT3 [4]. Since T3 is an active thyroid hormone but not T4, FT3/FT4, a marker of peripheral deiodination, also indicates thyroid hormone activity. 

Recently, thyroid hormones were shown to have a beneficial influence of preventing renal in jury [5]. We hypothesize that FT3/FT4 could be inversely associated with renal dysfunction, known as chronic kidney disease (CKD). 

In addition, a previous case-control study revealed that compared to people with euthyroidism, people with subclinical hypothyroidism (SCH) have lower renal function [6]. 

Among participants with CKD, an inverse association between glomerular filtration rate (GFR) and hypothyroidism has been reported [7]. A study with a nationally representative sample of American adults reported that reduced GFR is associated with a higher prevalence of hypothyroidism and SCH [8]. Thus, these studies indicate that SCH could be positively associated with CKD.

However, SCH is generally diagnosed as having elevated thyroid stimulating hormone (TSH) within the normal range of thyroid hormones such as T3 and T4. Previously, we reported that serum concentrations of thyroid hormones and thyroid hormone demand are both important determinants of thyroid function [9]. Therefore, if TSH is above the normal range, high-normal thyroid hormone activity that meets the level of demand might be beneficial for preventing CKD. FT3/FT4 could be positively associated with SCH, which could be positively associated with thyroid hormone activity. Furthermore, high-normal thyroid hormone activity might have a beneficial effect on renal function [6]. SCH without CKD, but not SCH with CKD, could be positively associated with FT3/FT4, suggesting that thyroid hormone activity could have a beneficial influence on preventing CKD.

SCH accelerates endothelial dysfunction [10] which is an initial step in the development of renal dysfunction [11]. Since thyroid hormone regulates the activity of endothelial repair [12,13,14], endothelial maintenance activity might influence the association among FT3/FT4, SCH, and CKD.

Clarifying those associations can help clarify the mechanisms underlying the association between thyroid function and CKD. We conducted a cross-sectional study of 1724 Japanese aged 40–74 years who received annual health examinations in 2014.

## 2. Materials and Methods

### 2.1. Study Population

Written consent forms were used to ensure that participants understood the objectives of the study when obtaining informed consent. This study was approved by the ethics committee of the Nagasaki University Graduate School of Biomedical Sciences (project registration number 14051404).

In 2008, to prevent lifestyle-related disease, the Japanese Ministry of Health, Labour and Welfare began annual health check-ups for people aged 40–74 years [15]. The present study was based on data from such annual check-ups.

**Figure 1:**
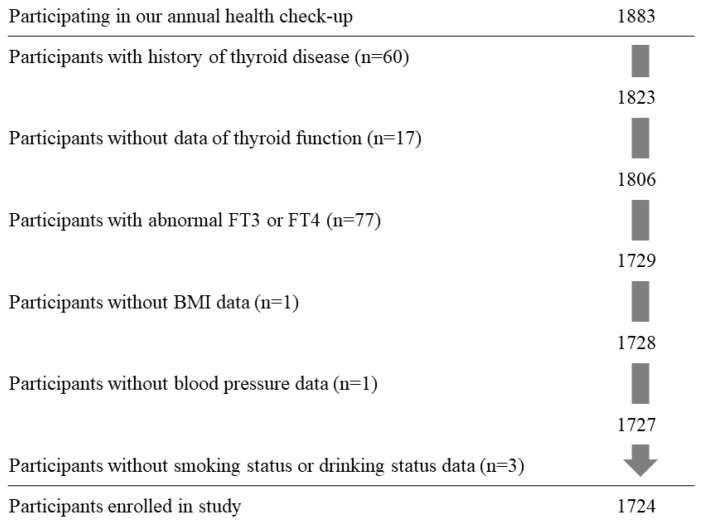
Demographic of study population. FT3: free triiodothyronine. FT4: free thyroxine. BMI: body mass index.

Figure 1 shows the demographic of the present study population. The study population comprised of 1883 Japanese individuals aged 40–74 years from Saza town in western Japan who underwent an annual medical examination in 2014. To preclude any influence of thyroid disease, participants with a history of thyroid disease (n = 60), missing data on thyroid function (TSH, FT3, or FT4) (n = 17), or abnormal FT3 (normal range, 2.1–4.1 pg/mL) or FT4 (normal range, 1.0–1.7 ng/dL) levels were excluded (n = 77). In addition, participants with missing data on body mass index (BMI) (n = 1), blood pressure (n = 1), or smoking status or drinking status (n = 3) were excluded. The remaining 1724 participants, with a mean age of 60.5 years (standard deviation (SD), 9.1 years; range, 40–74 years), were included in the study.

### 2.2. Data Collection and Laboratory Measurements

Methods used in the present study, including thyroid function evaluation, have been described elsewhere [9,16,17]. A trained interviewer obtained information on clinical characteristics, such as history of thyroid disease, glucose-lowering medication use, and smoking and drinking habits. Body weight and height were measured using an automatic body composition analyzer (BF-220; Tanita, Tokyo, Japan). BMI (kg/m^2^) was calculated. Systolic blood pressure (SBP) was recorded at rest.

Fasting blood samples were collected. TSH, FT3, and FT4 levels were measured using standard procedures at the LSI Medience Corporation (Tokyo, Japan). Glycohemoglobin (HbA1c), triglyceride (TG), high-density lipoprotein cholesterol (HDLc), and creatinine levels were measured using standard procedures at SRL Inc. (Tokyo, Japan). GFR was estimated using an established method with three variables proposed by a working group of the Japanese Chronic Kidney Disease Initiative [18]. 

The threshold for SCH was set at TSH > 4.01 μIU/mL. CKD was defined as GFR < 60 mL/min/1.73 m^2^.

### 2.3. Statistical Analysis

To evaluate the secretory capacity of the pituitary gland, i.e., sensitivity to thyroid hormone, Jostel’s TSH index was calculated [19]. Characteristics of the study population by FT3/FT4 tertile were expressed as means ± SDs, except for male sex, smoking status, drinking status, TSH, and TG. Male sex, smoking status, and drinking status were expressed as percentages. Since TSH and TG had skewed distributions, they were expressed as medians (interquartile range). Significant differences in FT3/FT4 levels were evaluated using analysis of variance.

Odds ratios (ORs) and 95% confidence intervals (CIs) were calculated using logistic regression to assess the association between SCH and CKD. Logistic regression was also used to calculate ORs and 95% CIs to determine the association between FT3/FT4 and SCH and the association between FT3/FT4 and CKD. To evaluate the association between FT3/FT4 and SCH by CKD status, we also calculate ORs and 95% CIs using logistic regression. 

SCH accelerates endothelial dysfunction [10]. Endothelial dysfunction is associated with most forms of cardiovascular disease, such as hypertension, coronary artery disease, chronic heart failure, peripheral artery disease, diabetes, and chronic renal failure [11]. Therefore, cardiovascular risk factors could be confounding factors in the present analysis.

Four adjustment models were used. Model 1 was adjusted for sex and age. Model 2 included the variables in Model 1 and potential confounding factors directly associated with thyroid function: TSH (μIU/mL). Model 3 was further adjusted for known cardiovascular risk factors indirectly associated with thyroid function: SBP (mmHg), BMI (kg/m^2^), drinking status (non, often, daily), smoking status (never, former, current), TG (mg/dL), HDLc (mg/dL), and HbA1c (%). Since TSH (μIU/mL) is not a confounding factor when evaluating the association between FT3/FT4 and SCH, we generated Model 4, which adjusted for sex, age, SBP (mmHg), BMI (kg/m^2^), drinking status (non, often, daily), smoking status (never, former, current), TG (mg/dL), HDLc level (mg/dL), and HbA1c (%).

All statistical analyses were performed using SAS for Windows, version 9.4 (SAS Inc., Cary, NC, USA). Results with *p* < 0.05 were considered statistically significant.

## 3. Results

Among 1724 study participants, 98 (5.7%) were diagnosed as having SCH and 302 (17.5%) were diagnosed with CKD.

### 3.1. Characteristics of the Study Population by FT3/FT4 Tertile

Table 1 shows the characteristics of the study population by FT3/FT4 tertile. FT3/FT4 was positively associated with FT3 and TSH, respectively. FT3/FT4 was inversely associated with FT4, Jostel’s TSH index, and TG, respectively. 

### 3.2. Association between SCH and CKD

Independent of known confounding factors, SCH was significantly positively associated with CKD (Table 2).

### 3.3. Associations between FT3/FT4 and CKD and between FT3/FT4 and SCH

FT3/FT4 was significantly inversely associated with CKD and significantly positively associated with SCH. These associations were unchanged even after further adjustment for known confounding factors (Table 3).

### 3.4. Association between FT3/FT4 and SCH with or without CKD

FT3/FT4 was significantly positively associated with SCH without CKD but not associated with SCH with CKD (Table 4).

## 4. Discussion

The major findings of the present study were that SCH is positively associated with CKD and FT3/FT4 is inversely associated with CKD while positively associated with SCH.

**Figure 2:**
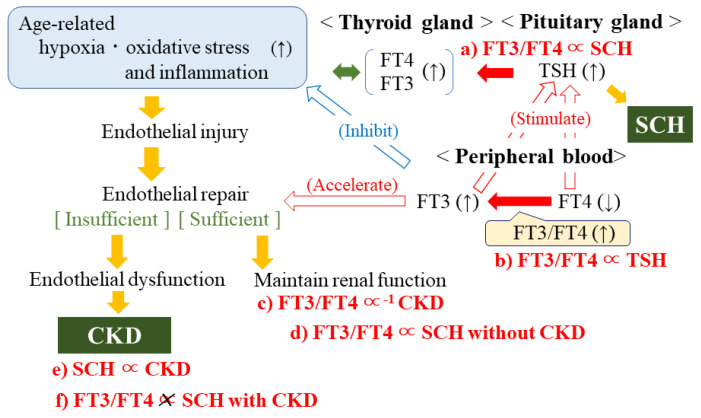
Potential mechanism underlying thyroid hormone and chronic kidney disease. Associations in red (**a**–**f**) were shown in present study. CKD: chronic kidney disease. FT3: free triiodothyronine. FT4: free thyroxine. TSH: thyroid stimulating hormone. SCH: subclinical hypothyroidism. Age-related physical changes such as increased oxidative stress, hypoxia, or inflammation injure the endothelium. Upon endothelial injury, endothelial repair is activated; insufficient endothelial repair results in progression of CKD. In peripheral blood, FT3/FT4 could act as a maker of endothelial repair activity by indicating thyroid hormone activity. In conjunction with the pituitary gland, the thyroid gland regulates the production of thyroid hormone.

Figure 2 shows the potential mechanisms underlying these findings. Associations in red (a–f) were shown in the present study. Positive associations among the prevalence of overt hypothyroidism, SCH, and progression of stage of CKD have been reported [20]. This profile is compatible with the results of our present study showing a significant positive association between SCH and CKD (Table 2, Figure 2e). Even within the normal range of thyroid hormone levels, low-normal thyroid hormone activity could be a risk factor for CKD. 

In the present study, we found further evidence that FT3/FT4 is significantly inversely associated with CKD and significantly positively associated with SCH. Since FT3 is an active thyroid hormone but not FT4, deiodinase activity, as reflected by FT3/ FT4, could indicate thyroid hormone activity even in individuals who are euthyroid. Therefore, in the present study, FT3/FT4 and FT3 were found to be positively associated (Table 1). This study also found that SCH is more common in individuals with high-normal thyroid hormone activity than in individuals with low-normal thyroid hormone activity. This seems contradictory because participants with SCH had higher thyroid hormone activity levels than those without SCH. 

The level of demand for endothelial repair might explain this contradiction. Thyroid hormone regulates the proliferation of endothelial progenitor cells [12], which are important in endothelial repair [13,14]. Therefore, thyroid hormone demands might be stronger in those who need endothelial repair. 

Since thyroid hormone shortage stimulates the production of TSH in the pituitary gland, the positive associations between FT3/FT4 and TSH (Table 1, Figure 2b) and between FT3/FT4 and FT3 (Table 1) indicate that individuals with high-normal thyroid hormone activity have higher thyroid hormone demand than individuals with low-normal thyroid hormone activity. Therefore, we found a significant positive association between FT3/FT4 and SCH (Table 3, Figure 2a). 

Since endothelial dysfunction is an initial step in the development of renal dysfunction, i.e., CKD [11], FT3/FT4 is significantly inversely associated with CKD, partly by reflecting levels of endothelial repair activity [12,13,14] and inhibition of the nuclear factor-kappa B pathway [5] (Table 3, Figure 2c).

Those mechanisms indicate that evaluating demand for thyroid hormone is a crucial part of evaluating thyroid function. Thyroid hormone demand can be evaluated as a ratio: actual thyroid hormone level/thyroid hormone demand. A detailed explanation of this concept was described elsewhere [9]. Participants with SCH in whom thyroid hormone productivity matches thyroid hormone demand might have a low risk of developing CKD. 

Therefore, SCH with insufficient adjustment for thyroid hormone demand might lead to CKD progression. Hypoglycemia, which is associated with high HbA1c, is reported to be associated with endothelial dysfunction [21]. Our previous prospective study with 2.8 years of follow-up found that higher HbA1c is associated with an annual decrease in renal function as evaluated by GFR in participants with SCH but not in those without SCH [22], which also provides support that insufficient adjustment for thyroid hormone demand might lead to CKD progression.

In addition, participants with SCH in whom thyroid hormone productivity cannot meet their thyroid hormone demands might have lower sensitivity to thyroid hormone. Low sensitivity to thyroid hormone is reflected by lower Jostel’s TSH index [19]. In the present study, even among participants with normal thyroid hormone levels, FT3/FT4 levels were inversely associated with Jostel’s TSH index (Table 1) and positively associated with SCH (Table 3). Since SCH is significantly positively associated with CKD (Table 2), our present findings are partly compatible with a previous study that reported a significant association between lower sensitivity to thyroid hormone and lower renal function among euthyroid individuals [23]. However, in the present study, we found a significant inverse association between FT3/FT4 levels and CKD (Table 3). This leads to a paradox because in our study, FT3/FT4 could be positively associated with low sensitivity to thyroid hormone while inversely associated with CKD. 

In individuals with euthyroidism, a positive association was reported between thyroid function evaluated by FT3 and renal function [24]. Another study of a patient with CKD reported a positive association between FT3 levels and GFR [25]. This study also reported an inverse association between FT4 and GFR. Since we found significant an inverse association between FT3/FT4 levels and CKD (Table 3), our present results are compatible with those studies. Therefore, the level of thyroid hormone demand, which is related to endothelial status but not sensitivity to thyroid hormone, might play an important role in the present results.

We found a significant positive association between FT3/FT4 and SCH without CKD (Table 4, Figure 2d). However, when TSH levels increased to meet the level of demand for thyroid hormones but thyroid hormone production was insufficient, as in participants with SCH and CKD, thyroid function might be relatively lower than in SCH participants without CKD. Since the level of demand for thyroid hormones could not be evaluated based on serum concentrations of thyroid hormones, no significant association was observed between FT3/FT4 and SCH with CKD (Table 4, Figure 2f).

The results of the present study have some clinical implications. The present study indicates that participants with SCH could have higher thyroid hormone levels but comparatively lower thyroid function than those without SCH because they have higher thyroid hormone demand. Therefore, actual thyroid function cannot be evaluated only based on serum thyroid hormone concentrations. High-normal thyroid activity could have a beneficial effect of preventing CKD. SCH could increase the beneficial influence of preventing CKD by increasing levels of thyroid hormones. 

Study limitations warrant consideration. Although levels of thyroid hormone demand play a prominent role in the present results, we have no effective methods to evaluate this demand level. Since thyroid hormone might act as a determinant of energy expenditure and basal metabolic rate [26], further investigation with data on mitochondrial metabolism [27,28] could be informative. Proliferation of endothelial progenitor cells [12] and inhibition of the nuclear factor-kappa B pathway [5] might play important roles in preventing CKD along with thyroid hormones, but we have no data on endothelial progenitor cells and nuclear factor-kappa B pathway activity. Further investigations with those data are required. In this cross-sectional study, causal relationships could not be established. However, we performed multifaceted analysis that enabled us to explore potential mechanisms to explain the present results. Glomerulonephritis, autosomal dominant polycystic kidney disease, some infections, and Alport syndrome might influence on the present results, but we have no data about those conditions. However, we believe that the influence of those diseases in the present study should be limited because our study is performed among the general population.

## 5. Conclusions

FT3/FT4, a marker of peripheral T4 deiodination, is positively associated with SCH and inversely associated with CKD. However, SCH is positively associated with CKD. Present study indicates that higher activity of peripheral thyroxin deiodination might have beneficial association on the absence of CKD, even among SCH which is positively associated with CKD. 

## Figures and Tables

**Table 1:** Characteristics of study population in relation to FT3/FT4.

	FT3/FT4 Levels (Tertile)			*p*
	Low	Middle	High	
No. of participants	573	577	574	
Men, %	37.3	36.4	37.3	0.933
Age	60.6 ± 9.4	60.2 ± 9.1	60.7 ± 9.0	0.552
FT3, pg/mL	3.0 ± 0.3	3.2 ± 0.3	3.3 ± 0.3	<0.001
FT4, ng/dL	1.4 ± 0.1	1.2 ± 0.1	1.1 ± 0.1	<0.001
TSH, μIU/mL	1.50 [1.05, 2.22] *^1^	1.57 [1.12, 2.24] *^1^	1.64 [1.11, 2.39] *^1^	0.046 *^2^
Jostel’s TSH index	2.80 ± 0.64	2.61 ± 0.60	2.45 ± 0.60	<0.001
BMI, kg/m^2^	22.7 ± 3.5	22.7 ± 3.3	22.9 ± 3.3	0.489
Daily drinker, %	42.9	39.0	39.0	0.295
Non-drinker, %	53.1	58.4	57.3	0.155
Current smoker, %	12.9	13.9	14.3	0.787
Former smoker, %	22.2	20.8	19.7	0.586
Systolic blood pressure, mmHg	125 ± 16	125 ± 16	125 ± 18	0.789
TG, mg/dL	92 [65, 131] *^1^	84 [63, 123] *^1^	85 [63, 119] *^1^	0.026 *^2^
HDLc, mg/dL	61 ± 17	60 ± 14	60 ± 14	0.221
HbA1c, %	5.7 ± 0.7	5.6 ± 0.6	5.6 ± 0.6	0.058

FT3, free triiodothyronine; FT4, free thyroxine; TSH, thyroid stimulating hormone; TG, Triglycerides; HDLc, HDL-cholesterol; HbA1c, glycohemoglobin. Values are mean ± standard deviation. *^1^: Values are median (the first quartile, the third quartile). *^2^: Logarithmic transformation was used for evaluating p. Tertile values of FT3/FT4 ratios for men were <2.43 [pg × 10^2^/ng] for low, 2.43–2.70 [pg × 10^2^/ng] for middle, and 2.71 [pg × 10^2^/ng] ≤ for high. The corresponding values for women were <2.40 [pg × 10^2^/ng], 2.40–2.69 [pg × 10^2^/ng], and 2.70 [pg × 10^2^/ng].

**Table 2:** Association between subclinical hypothyroidism (SCH) and chronic kidney disease (CKD).

	Subclinical Hypothyroidism (SCH)		*p*
	(–)	(+)	
No. at risk	1626	98	
**Chronic kidney disease (CKD)**			
No. of case (%)	272 (16.7)	30 (30.6)	
Model 1	Ref	2.10 (1.31, 3.35)	0.002
Model 4	Ref	2.23 (1.38, 3.59)	0.001

Model 1: adjusted only for sex and age. Model 4: Adjusted for sex, age, systolic blood pressure (SBP), body mass index (BMI), drinking status, smoking status, triglycerides (TG), high-density lipoprotein cholesterol (HDLc), glycohemoglobin (HbA1c).

**Table 3:** Association between FT3/FT4 and chronic kidney disease (CKD), subclinical hypothyroidism (SCH).

	FT3/FT4 Levels (Tertile)			*p*	1 SD Increment of FT3/FT4
	Low	Middle	High		
No. at risk	573	577	574		
**Subclinical hypothyroidism (SCH)**					
No. of case (%)	24 (4.2)	33 (5.7)	41 (7.1)		
Model 1	Ref	1.40 (0.82, 2.40)	1.76 (1.05, 2.95)	0.032	2.33 (1.31, 4.13)
Model 4	Ref	1.52 (0.87, 2.64)	1.92 (1.12, 3.26)	0.018	2.40 (1.34, 4.29)
**Chronic kidney disease (CKD)**					
No. of case (%)	125 (21.8)	88 (15.3)	89 (15.5)		
Model 1	Ref	0.66 (0.48, 0.89)	0.64 (0.47, 0.87)	0.004	0.58 (0.40, 0.83)
Model 2	Ref	0.64 (0.47, 0.87)	0.60 (0.44, 0.82)	0.001	0.52 (0.36, 0.75)
Model 3	Ref	0.64 (0.46, 0.87)	0.58 (0.42, 0.80)	<0.001	0.51 (0.35, 0.74)

Model 1: adjusted only for sex and age. Model 2: further adjusted (Model 1 +) for thyroid stimulating hormone (TSH). Model 3: further adjusted for (Model 2+) systolic blood pressure (SBP), body mass index (BMI), drinking status, smoking status, triglycerides (TG), high-density lipoprotein cholesterol (HDLc), glycohemoglobin (HbA1c). Model 4: Adjusted for sex, age, SBP, BMI, drinking status, smoking status, TG, HDLc, HbA1c. Tertile values of FT3/FT4 ratios for men were <2.43 [pg × 10^2^/ng] for low, 2.43–2.70 [pg × 10^2^/ng] for middle, and 2.71 [pg × 10^2^/ng] ≤ for high. The corresponding values for women were <2.40 [pg × 10^2^/ng], 2.40–2.69 [pg × 10^2^/ng], and 2.70 [pg × 10^2^/ng].

**Table 4:** Association between FT3/FT4, subclinical hypothyroidism (SCH) with or without chronic kidney disease (CKD).

	FT3/FT4 Levels (Tertile)			*p*	1 SD Increment of FT3/FT4
	Low	Middle	High		
No. at risk	573	577	574		
**Subclinical hypothyroidism (SCH) without chronic kidney disease (CKD)**					
No. of case (%)	12 (2.1)	26 (4.5)	30 (5.2)		
Model 1	Ref	2.20 (1.10, 4.41)	2.58 (1.31, 5.09)	0.007	3.17 (1.60, 6.30)
Model 4	Ref	2.54 (1.24, 5.20)	2.98 (1.47, 6.05)	0.003	3.44 (1.72, 6.91)
**Subclinical hypothyroidism (SCH) with chronic kidney disease (CKD)**					
No. of case (%)	12 (2.1)	7 (1.2)	11 (1.9)		
Model 1	Ref	0.60 (0.23, 1.53)	0.92 (0.40, 2.12)	0.839	1.16 (0.43, 3.17)
Model 4	Ref	0.59 (0.23, 1.54)	0.91 (0.39, 2.14)	0.833	1.11 (0.40, 3.06)

FT3, free triiodothyronine; FT4, free thyroxine. Model 1: adjusted only for sex and age. Model 4: Adjusted for sex, age, systolic blood pressure (SBP), body mass index (BMI), drinking status, smoking status, triglycerides (TG), high-density lipoprotein cholesterol (HDLc), glycohemoglobin (HbA1c). Tertile values of FT3/FT4 ratios for men were <2.43 [pg × 10^2^/ng] for low, 2.43–2.70 [pg × 10^2^/ng] for middle, and 2.71 [pg × 10^2^/ng] ≤ for high. The corresponding values for women were <2.40 [pg × 10^2^/ng], 2.40–2.69 [pg × 10^2^/ng], and 2.70 [pg × 10^2^/ng].

## Data Availability

We cannot publicly provide individual data due to participant privacy, according to ethical guidelines in Japan. Additionally, the informed consent obtained does not include a provision for publicly sharing data. Qualifying researchers may apply to access a minimal dataset by contacting Prof. Naomi Hayashida, Principal Investigator, Division of Promotion of Collaborative Research on Radiation and Environment Health Effects, Atomic Bomb Disease Institute, Nagasaki University, Nagasaki, Japan at naomin@nagasaki-u.ac.jp. Or, please contact the office of data management at ritouken@vc.fctv-net.jp. Information for where data requests are also available are at https://www.genken.nagasaki-u.ac.jp/dscr/message/ (accessed on 20 December 2021) and http://www.med.nagasaki-u.ac.jp/cm/ (accessed on 20 December 2021).
